# Supplementary material for: Investigation of Astyanax mexicanus (Characiformes, Characidae) chromosome 1 structure reveals unmapped sequences and suggests conserved evolution
Source: PLoS One. 2024 Nov 18;19(11):e0313896. doi: 10.1371/journal.pone.0313896 (PMC11573200; doi:10.1371/journal.pone.0313896)
Supplement: S1 Table — (DOCX) [file pone.0313896.s001.docx]

**Table 1** – Primers used for amplification of gene fragments present on chromosome 1

| **Gene** | **Primer** | **Size** |
| --- | --- | --- |
| **Nyap1** | F- TGTCCTTCTCTCCTCCCGAG  R- CCCAAAGCTACCGTGTCCAT | 382 bp  (Region Cr1: 373,336 – 373,717) |
| **Mllt10** | F- ACATGGGCCGTATCGATTCC  R-GCACTGACTCCTACACCACC | 341 bp  (Region Cr1: 27,312,307 – 27,312,620) |
| **Pdcb** | F- GCTTTGGGCCCAAGTTTGAG  R-AGGGAGCAGTCCGTAGTCAT | 368 bp  (Region Cr1: 73,882,992 – 73,883,359) |
