# Supplementary material for: Investigation of Astyanax mexicanus (Characiformes, Characidae) chromosome 1 structure reveals unmapped sequences and suggests conserved evolution
Source: PLoS One. 2024 Nov 18;19(11):e0313896. doi: 10.1371/journal.pone.0313896 (PMC11573200; doi:10.1371/journal.pone.0313896)
Supplement: S2 Table — SatA, SatB, SatC, and SatD are present on chromosome 1. The satellite sequences SatE and SatF are outside of chromosome 1 and were used to validate the microdissected chromosome. (DOCX) [file pone.0313896.s002.docx]

**Table 2 –** Primers used for amplification of Satellite DNA sequences. SatA, SatB, SatC, and SatD are present on chromosome 1. The satellite sequences SatE and SatF are outside of chromosome 1 and were used to validate the microdissected chromosome.

| **Satellite DNA** | **Primer** | **Size** |
| --- | --- | --- |
| SatA_mex | F - TGCATCTCGGTGGACAACAA  R - GCAACATTCAGCCTGCCAAA | 574 bp |
| SatB_mex | F - TGTGGGTCAGTGTGTGTTCC  R - ACCCACACAGTGTCATGTGA | 583 bp |
| SatC_mex | F- GAGTGGGAAAATGCATGGGC  R- GCATTTTCCCACTCGTTTGA | 179 bp |
| SatD_mex | F - CTGCCTAGCAACCACTTGGA  R - GGCAGTTGCTAAGTGGTTGC | 54 bp |
| SatE_mex | F - CCGTTGTGGTAGTATGTGCC  R – ACGGTTCTGCCATCGTTTGA | 152 bp |
| SatF_mex | F - TGCCGAATCCTAGCCACTAG  R – TAGGATTCGGCACTCTCACC | 656 bp |
