# Supplementary material for: Investigation of Astyanax mexicanus (Characiformes, Characidae) chromosome 1 structure reveals unmapped sequences and suggests conserved evolution
Source: PLoS One. 2024 Nov 18;19(11):e0313896. doi: 10.1371/journal.pone.0313896 (PMC11573200; doi:10.1371/journal.pone.0313896)
Supplement: S3 Table — (DOCX) [file pone.0313896.s003.docx]

**Table 3 -** Similarity with satDNAs described in the literature.

| Satellite | Size | Hit | Genbank Access | Coverage (%) | Identity (%) |
| --- | --- | --- | --- | --- | --- |
| SatA_mex | 1819 | PliSat13-1928 | MZ161106.1 | 71 | 69,02 |
|  |  | CmoSat070-776 | OR604437.1 | 10 | 82,54 |
|  |  | CmoSat013-2520 | OR604380.1 | 14 | 70,61 |
|  |  | PseSat08-2005 | OR094708.1 | 9 | 72,99 |
|  |  | Cmasat23-898 | OP784504.1 | 11 | 73,29 |
| SatB_mex | 583 | AlaSat28-574 | OM793274.1 | 83 | 85,98 |
| SatC_mex | 179 | AlaSat02-186 | OM793249.1 | 97 | 88,4 |
|  |  | ApaSat10-179 | MF044776.1 | 100 | 95,41 |
| SatD_mex | 54 | PfaSat03-97 | OM793194.1 | 98 | 92,31 |
| SatE_mex | 152 | AlaSat04-151 | OM793251.1 | 57 | 87,33 |
|  |  | PfaSat27-197 | OM793218.1 | 76 | 70,31 |
| SatF_mex | 656 | PboSat31-657 | OM793173.1 | 100 | 97,67 |
|  |  | AlaSat31-552 | OM793277.1 | 83 | 96,47 |
|  |  | Cmasat40-932 | OP784521.1 | 35 | 80,25 |
|  |  | CmoSat065-695 | OR604432.1 | 23 | 100 |
|  |  | PliSat39-915 | MZ161131.1 | 23 | 95,45 |
