# Supplementary material for: Investigation of Astyanax mexicanus (Characiformes, Characidae) chromosome 1 structure reveals unmapped sequences and suggests conserved evolution
Source: PLoS One. 2024 Nov 18;19(11):e0313896. doi: 10.1371/journal.pone.0313896 (PMC11573200; doi:10.1371/journal.pone.0313896)
Supplement: S4 Table — (DOCX) [file pone.0313896.s004.docx]

**Table 4 –** Main scaffolds with reads anchored and not yet assigned to chromosomes

| **Scaffold** | **Anchoring region of reads** |
| --- | --- |
| Chromosome NW_019171364.1 | 1004000000 - 1005500000 |
| Chromosome NW_019171377.1 | 1007765000 - 1007785000 |
| Chromosome NW_019171397.1 | 1008980000 - 1009010000 - 1009040000 |
| Chromosome NW_019171399.1 | 1009150000 - 1009250000 |
| Chromosome NW_019171483.1 | 1015250000 – 1015350000 - |
| Chromosome NW_019171491.1 | 1015800000 - 1016000000 - 1016200000 |
| Chromosome NW_019171635.1 | 1029200000 - 1029800000 - 1030400000 |
| Chromosome NW_019171672.1 | 1033900000 - 1034100000 - 1034300000 |
| Chromosome NW_019171699.1 | 1037020000 - 1037050000 - 1037080000 |
| Chromosome NW_019171704.1 | 1037400000 - 1037600000 - 1037800000 |
| Chromosome NW_019171784.1 | 1046280000 - 1046340000 - 1046400000 |
| Chromosome NW_019172066.1 | 1065550000 - 1065700000 - 1065850000 |
| Chromosome NW_019172086.1 | 1067500000 - 1067600000 - 1067700000 |
| Chromosome NW_019172134.1 | 1071450000 - 1071550000 - 1071650000 |
